# Epoxyeicosatrienoic Acids Attenuating Hypotonic-Induced Apoptosis of IMCD Cells via γ-ENaC Inhibition

**DOI:** 10.1371/journal.pone.0094400

**Published:** 2014-04-08

**Authors:** Luyun Wang, Yang Liu, Huamin Wang, Xun Liu, Jie Chen, Mong-Heng Wang, Jingfeng Wang, Hui Huang

**Affiliations:** 1 Guangdong Province Key Laboratory of Arrhythmia and Electrophysiology, Guangzhou, China; 2 Department of Cardiology, Sun Yat-sen Memorial Hospital of Sun Yat-sen University, Guangzhou, China; 3 Department of Critical Care Medicine, Sun Yat-sen Memorial Hospital of Sun Yat-sen University, Guangzhou, China; 4 Zhongshan City Hospital of Chinese Medicine,Affiliated Hospital of Guangzhou University of Chinese Medicine, Zhongshan, China; 5 Division of Nephrology, Department of Internal Medicine, The Third Affiliated Hospital of Sun Yat-sen University, Guangzhou, China; 6 Radiotherapy Department, Sun Yat-sen Memorial Hospital of Sun Yat-sen University, Guangzhou, China; 7 Department of Physiology, Georgia Regents University, Augusta, Georgia, United States of America; Emory University, United States of America

## Abstract

Inner medulla collecting duct (IMCD) cells are the key part for urinary concentration. Hypotonic stress may trigger apoptosis of IMCD cells and induce renal injury. Epoxyeicosatrienoic acids (EETs) play an important role in anti-apoptosis, but their roles in hypotonic-induced apoptosis of IMCD cells are still unclear. Here we found increasing exogenous 11, 12-EET or endogenous EETs with Ad-CMV-CYP2C23-EGFP transfection decreased apoptosis of IMCD cells induced by hypotonic stress. Moreover, up-regulation of γ-ENaC induced by hypotonic stress was abolished by elevation of exogenous or endogenous EETs. Collectively, this study illustrated that EETs attenuated hypotonic-induced apoptosis of IMCD cells, and that regulation of γ-ENAC may be a possible mechanism contributing to the anti-apoptotic effect of EETs in response to hypotonic stress.

## Introduction

The kidney maintains stabilized extracellular volume and osmolality in body predominantly through electrolyte and water reabsorption. Inner medulla collecting duct (IMCD) is the final part of collecting duct, together with cortical collecting duct, contributing to reabsorbing 4–5% of filtered sodium. Sodium enters principal cells of IMCD through epithelial sodium channel (ENaC) [1]. ENaC consists of three homologous subunits, α, β and γ, which share 35% homology in amino acid sequence, among these three subunits, γ-ENaC is the major subunit responsible for sodium absorption in IMCD [2], [3]. From diuresis to antidiuresis, IMCD cells are constantly subjected to varying osmolar conditions [1]. However, severe osmotic stress exceeding the compensative capacity of cells may trigger a self-destruction program to programmed cell death (apoptosis) [4]. ENaC, as a rate-limiting step in transepithelial sodium reabsorption, has been involved in the process [5]–[8].

Epoxyeicosatrienoic acids (EETs) are metabolites of arachidonic acid (AA) by cytochrome P450 (CYP) epoxygenases, including four regioisomers: 5, 6-, 8, 9-, 11, 12-, and 14, 15-EET. These EETs are synthesized mainly by epoxygenases of the CYP2C and CYP2J family [9]. It is now widely accepted that CYP2C23 is the predominant and functionally relevant epoxygenase isoform in rat kidney. It is mainly responsible for 11, 12-EET formation [10]. EETs exhibit diverse and critical biological effects in renal protection, including regulating renal circulation [11]–[13], anti-inflammation, anti-thrombosis [14], increasing cell survival and decreasing apoptosis [15]–[19].

Sustained hypotonic stress in luminal and interstitial surroundings challenges the survival of IMCD cells[20]. Although, previous researches demonstrated that EETs are anti-apoptotic in several cases [9], [19], [21], the role of EETs in IMCD cells is not clear. Do EETs protect IMCD cells from the challenges of sustained hypotonic stress? The present studies were designed to determine the effects of EETs on IMCD cells exposed to hypotonic stress. Furthermore, we determined whether the effects of EETs on the apoptosis of IMCD cells under hypotonic stress were mediated through γ-ENaC, as one of the possible mechanisms.

## Results

### Hypotonic stress inhibited proliferation and induced apoptosis in IMCD cells

**Figure 1:**
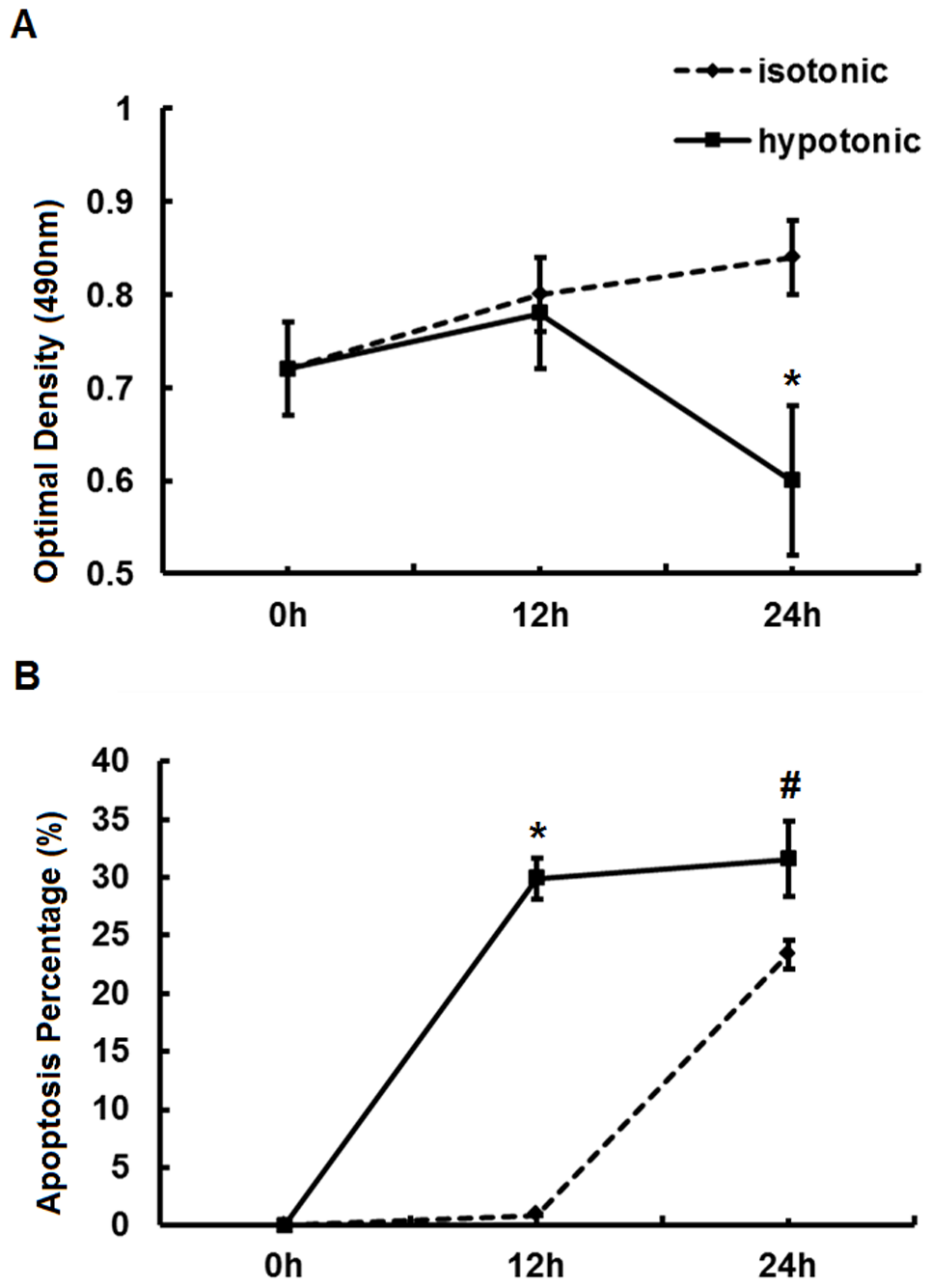
Proliferation and apoptosis assessed by MTT assay and FACS analysis respectively. (A) MTT assay assessing the proliferation of IMCD cells exposed to hypotonic or isotonic stress for 0 h, 12 h and 24 h. (B) FACS assessing the apoptosis percentage of IMCD cells exposed to hypotonic or isotonic stress for 0 h, 12 h and 24 h. *, *P*<0.05 compared with isotonic group. #, *P*<0.05 compared with isotonic group. Error bars represent the standard deviations based on three independent replicates.

To evaluate the effects of hypotonic stress on IMCD cells, MTT and FACS analysis were performed. As shown in Fig. 1A, B, the proliferation of IMCD cells began to decrease after being exposed to hypotonic stress for 12 h (hours), but the decrease in proliferation rate was not significant until the 24th hour (0.6±0.08 *vs*. 0.84±0.04, *P*<0.05). Meanwhile, flow cytometry analysis (FACS) results showed that the apoptosis percentage increased significantly after being exposed to hypotonic stress 12 h compared to isotonic group (29.9%±1.8% *vs*. 0.9%±0.08%, *P*<0.05). The apoptosis percentage was further increased after extending exposure time to 24 h (31.6%±3.28% *vs*. 23.4%±1.24%, *P*<0.05). These results illustrated that hypotonic stress inhibited proliferation and induced apoptosis of IMCD cells in a time-dependent manner.

### Overexpression of CYP2C23 by adenovirus in IMCD cells

**Figure 2:**
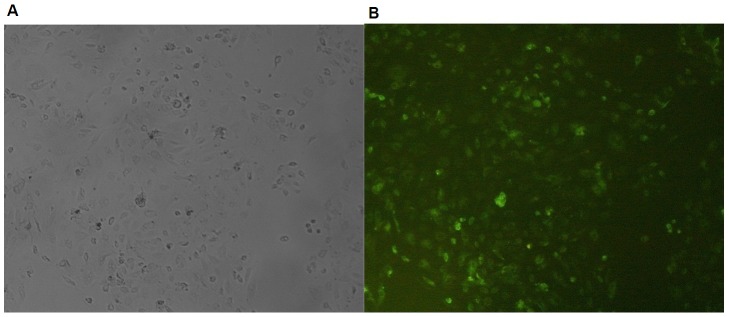
The fluorescence intensity in IMCD cells transfected with adenovirus vectors. IMCD cells were transfected with vehicle adenovirus vectors and Ad-CMV-CYP2C23-EGFP (green) at MOI of 100. Fluorescence intensity was observed at 24 h post-transfection (×10). (A) Vehicle adenovirus vectors. (B) Ad-CMV-CYP2C23-EGFP.

To demonstrate the transfection efficiency of recombinant CYP2C23, we observed the green fluorescence in IMCD cells transfected with Ad-CMV-CYP2C23-EGFP and adenovirus vectors without CYP2C23-EGFP (vehicle vectors) at MOI of 100. The transfection yielded marked GFP protein expression (green fluorescence) in IMCD cells, but not in vehicle-treated cells (Fig. 2). The transfection efficiency was 80.35%±3.23% at 24 h. It indirectly indicated the overexpression of CYP2C23 and elevation of CYP2C23-mediated EETs in IMCD cells.

### EETs alleviated the inhibition of IMCD cells proliferation induced by hypotonic stress

**Figure 3:**
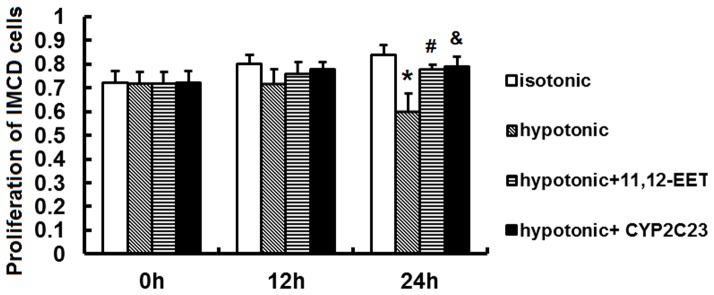
Proliferation assessed by MTT assay. The results of MTT assay on assessing the effect of 11,12-EETs (100 ng/ml) or Ad-CMV-CYP2C23-GFP (100 MOI) transfection on the proliferation of IMCD cells exposed to 0 h,12 h or 24 h hypotonic stress. *, *P*<0.05 compared with isotonic group. #, *P*<0.05 compared with hypotonic group. &, *P*<0.05 compared with hypotonic group. Error bars represent the standard deviations based on three independent replicates.

To evaluate the effects of exogenous and endogenous EETs on IMCD cells proliferation exposed to hypotonic stress, we conducted experiments involving either addition of 11,12-EETs or elevation of endogenous EETs via overexpression of CYP2C23 by Ad-CMV-CYP2C23-EGFP, and then determined the proliferative effects of hypotonic stress on IMCD cells by MTT viability assays. As shown in Fig. 3, hypotonic stress caused a time-dependent decrease in the proliferation of IMCD cells compared with isotonic stress. Exogenous 11,12-EET (100 ng/ml) significantly attenuated the inhibition of cell proliferation induced by 24 h hypotonic stress exposure. Increasing endogenous EETs by Ad-CMV-CYP2C23-EGFP transfection showed similar effects on IMCD cells. These findings indicated that CYP epoxygenase-mediated EETs promoted the proliferation of IMCD cells.

### EETs prevented the apoptosis of IMCD cells induced by hypotonic stress

**Figure 4:**
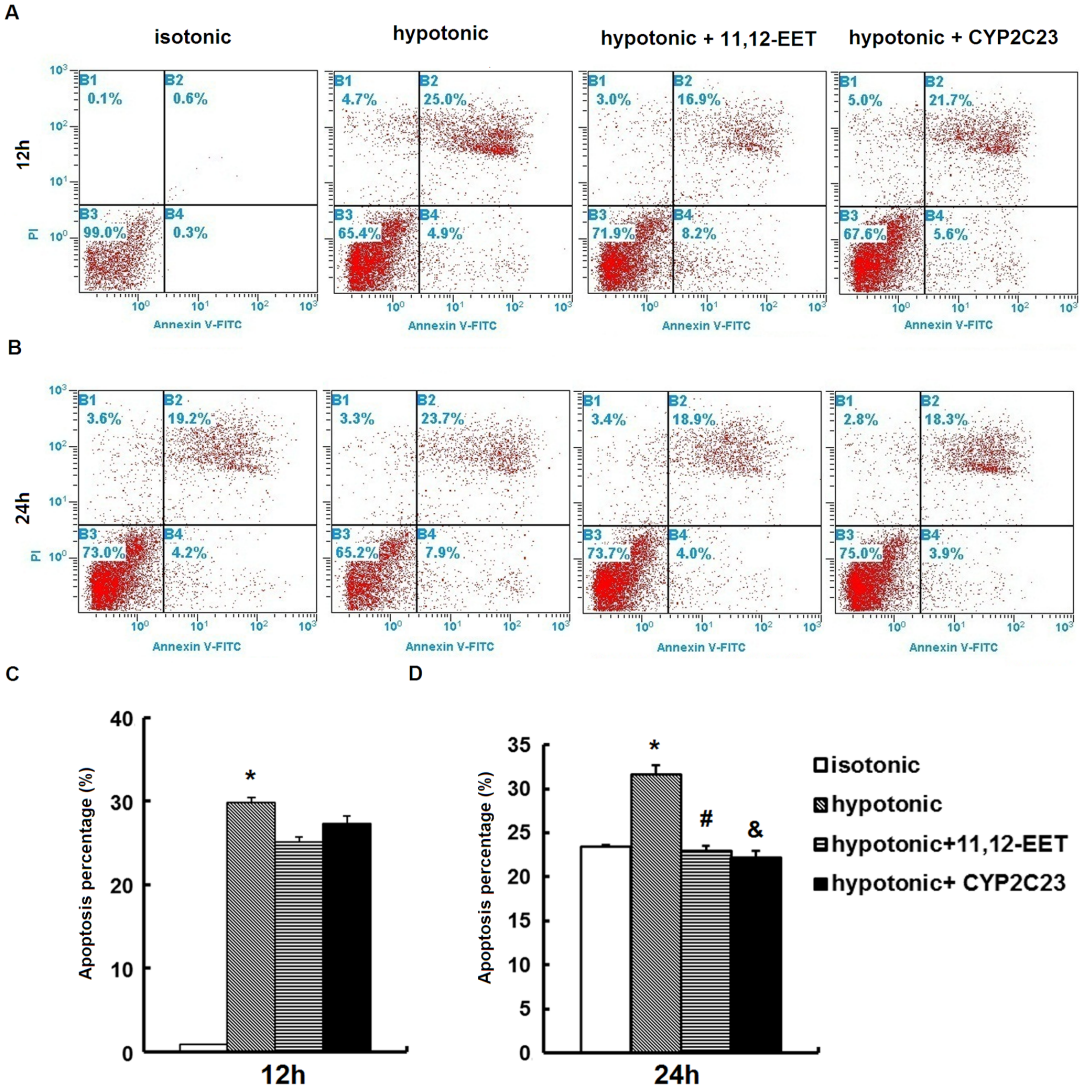
Apoptosis assessed by FACS analysis. Representative figures and results of FACS analysis on the effect of 11,12-EETs (100 ng/ml) or Ad-CMV-CYP2C23-GFP (100 MOI) transfection on IMCD cells apoptosis induced by 12 h or 24 h hypotonic stress. (A) (C) 11,12-EETs or Ad-CMV-CYP2C23-GFP transfection showed an inhibitory but non-significant effect on the apoptosis of IMCD cells induced by 12 h hypotonic stress. (B) (D) 11,12-EETs or Ad-CMV-CYP2C23-GFP transfection significantly inhibited the apoptosis of IMCD cells induced by 24 h hypotonic stress. *, *P*<0.05 compared with isotonic group. #, *P*<0.05 compared with hypotonic group. &, *P*<0.05 compared with hypotonic group. Error bars represent the standard deviations based on three independent replicates.

As mentioned above, treatment with 11,12-EET or Ad-CMV-CYP2C23-EGFP transfection in IMCD cells recovered the inhibition of proliferation induced by hypotonic stress, suggesting potential anti-apoptotic effect of EETs. For further investigation, apoptosis was determined by annexin-V-FITC-PI analysis of IMCD cells exposed to hypotonic stress using flow cytometry. Treatment with 11,12-EET resulted in a moderate decrease in the apoptosis percentage of IMCD cells exposed to 12 h of hypotonic stress (25.10%±2.49% *vs*. 29.90%±1.8%, *P*>0.05), indicating a possible anti-apoptotic effect of 11,12-EET. Similarly, Ad-CMV-CYP2C23-EGFP transfection also decreased apoptosis (27.30%±3.34% *vs*. 29.90%±1.8%, *P*>0.05) (Fig. 4A, C). The apoptosis percentage of IMCD cells exposed to hypotonic stress for 24 h was steadily alleviated by treatment with exogenous 11,12-EET or Ad-CMV-CYP2C23-EGFP transfection (22.90%±2.56%, 22.20%±3.13% *vs*. 31.60%±3.28%, *P*<0.05) (Fig. 4B, D). The results demonstrated that CYP epoxygenases and their eicosanoid metabolites EETs prevented the apoptosis of IMCD cells induced by hypotonic stress in a time-dependent manner.

### EETs inhibited the up-regulation of γ-ENaC induced by hypotonic stress

**Figure 5:**
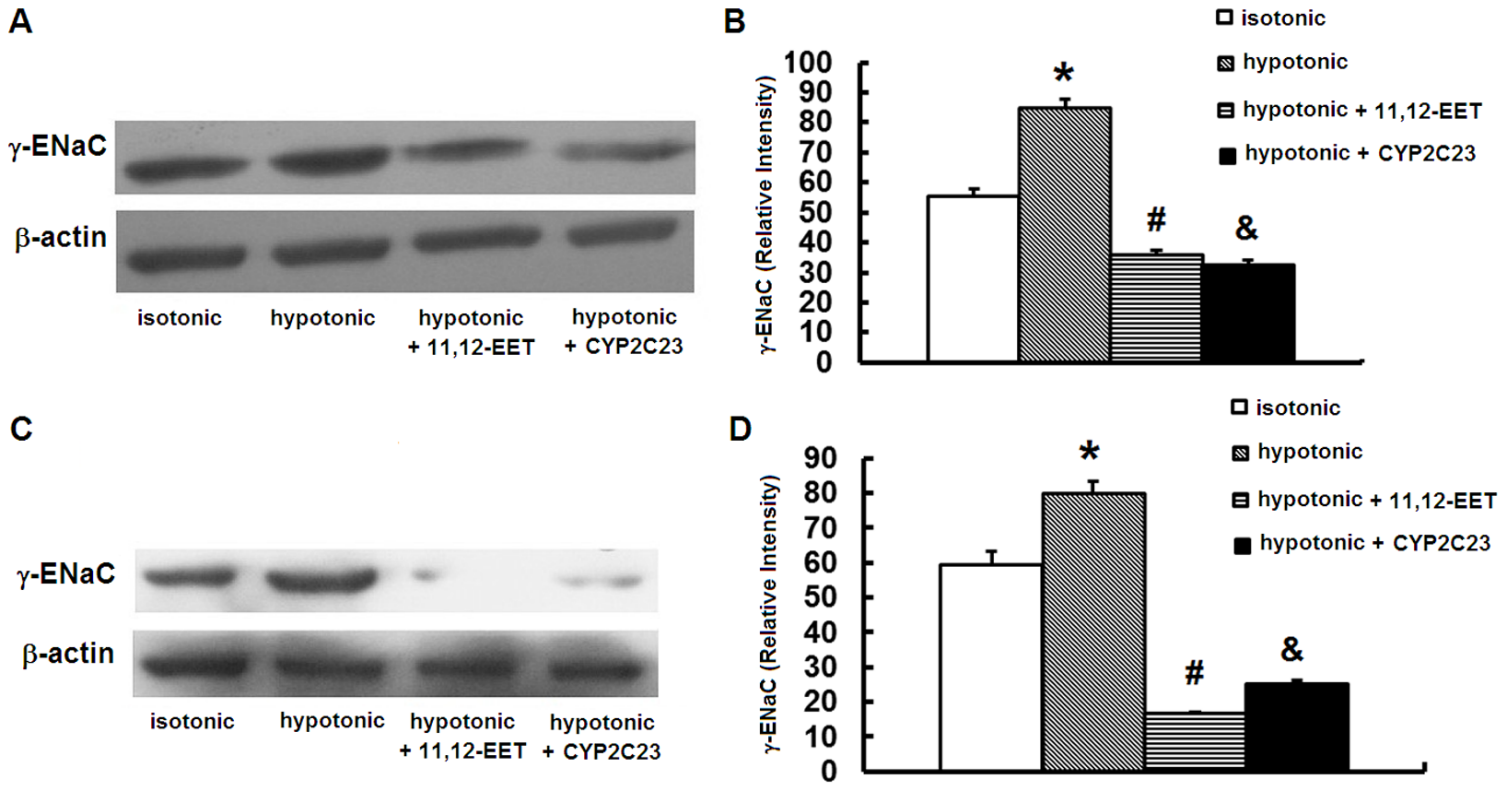
Protein γ-ENaC detected by western blot. EETs and CYP2C23 transfection decreased apoptosis of IMCD cells induced by hypotonic stress via γ-ENaC. (A)(B) EETs or Ad-CMV-CYP2C23-EGFP transfection inhibited the up-regulation of γ-ENaC induced by 12 h hypotonic stress. (C) (D) EETs or Ad-CMV-CYP2C23-EGFP transfection inhibited the up-regulation of γ-ENaC induced by 24 h hypotonic stress. (A) (C) Representative blots. (B) (D) Densitometric analysis. *, *P*<0.05 compared with isotonic group. #, *P*<0.05 compared with hypotonic group. &, *P*<0.05 compared with hypotonic group. Error bars represent the standard deviations based on three independent replicates.

To explore whether γ-ENaC is involved in the anti-apoptotic effects of EETs, we determined the protein level of γ-ENaC in IMCD cells exposed to hypotonic stress in absence and presence of 11,12-EET or Ad-CMV-CYP2C23-EGFP transfection. We found that hypotonic stress increased γ-ENaC expression as well as the apoptosis in IMCD cells at 12 h and 24 h. At both time points, treatment with 11,12-EET attenuated the elevation of γ-ENaC protein expression while decreased the apoptosis of IMCD cells. Ad-CMV-CYP2C23-EGFP transfection produced similar results at both time points (Fig. 5A, B, C, D). These data indicated that up-regulation of γ-ENaC could be induced by hypotonic stress, and that increasing exogenous and endogenous EETs inhibited the up-regulation of γ-ENaC, which suggested the involvement of γ-ENaC in the anti-apoptotic role of EETs in IMCD cells exposed to hypotonic stress.

## Discussion

In the present study, we showed that hypotonic stress inhibited proliferation and induced apoptosis in IMCD cells. Endogenous or exogenous EETs exhibited anti-apoptotic and pro-proliferative effects on the IMCD cells exposed to hypotonic stress. Down-regulation of γ-ENaC may be one of the possible mechanisms contributing to the process.

In the present study, IMCD cells were exposed to a hypotonic culture medium (200 mOsmol/kgH_2_O) composed of NaCl for 12 h and 24 h. Extremely low concentration of sodium results in hypotonic stress, challenging the survival of IMCD cells. Previous work showed that hypotonic stress significantly lowered the viability of mIMCD3 cells (an established cell line of inner medulla collecting duct derived from murine) [22]. Here, we further demonstrated that hypotonic stress inhibited proliferation and induced apoptosis of IMCD cells in vitro.

EETs show anti-apoptotic effects in some cases. Overexpression of CYP2J2 gene by recombinant adeno-associated virus (rAAV) delivery protects remnant kidney against renal injury in 5/6-Nx rats by inhibiting apoptosis [19]. 14,15-EET inhibits the apoptosis in LLCPKcl4 (an established adherent proximal tubule-like epithelial cell line derived from pig kidney) induced by serum starvation, H_2_O_2,_ or etoposide [9]. The sEH-deficient mice with an elevated level of endogenous EETs show decreased renal tubular apoptosis induced by diabetes [23]. In the present research, treatment with exogenous 11, 12-EET or increasing endogenous EETs by overexpression of CYP2C23 showed significant pro-proliferative and anti-apoptotic effects on the IMCD cells exposed to hypotonic stress. Our results added new evidences for the anti-apoptotic role of EETs in renal protection.

The mechanisms involved in the anti-apoptotic effects of EETs appear to differ depending on species, type of cell, and EET regioisomers [9], [15], [17], [19], [23]. EETs have been proven to inhibit the expression and activity of ENaC. In polarized M1 cells (derived from mouse lymphocytes), 14, 15-EET inhibits ENaC, which is associated with increased threonine phosphorylation of the ENaC β and γ subunits [24]. CYP-epoxygenase metabolites of AA, mainly 11,12-EET, inhibits ENaC in cortical collecting duct. Down-regulation of CYP2C44 abolishes AA-mediated inhibition of ENaC [10], [13], [25]. What's more, ENaC has been shown to be involved in the process of apoptosis. For example, α-ENaC is involved in the apoptosis of primary rat alveolar epithelial cells induced by hypoxia [6], [8], as well as the apoptosis of HepG2 (a perpetual cell line derived from hepatocellular carcinoma) [7]. In this study, we observed that hypotonic stress increased γ-ENaC expression in IMCD cells, which was abolished by endogenous and exogenous EETs.

Nevertheless, how ENaC regulated apoptosis is still unclear. Electrochemical gradients change of monovalent ions across cell membrane lead to cell volume perturbations, which ultimately result in cell death [26]. Marunaka, Y et al. reported that up-regulation of ENaC increased Na+ influx paralleling with Cl-, the cell volume would increase associated with an increase in cytosolic Cl- concentration[27]-[29]. Admittedly, contrasting cell volume behaviors (shrinkage vs. swelling) are features that commonly serve to distinguish apoptosis from necrosis, but cell type-specific rather than ubiquitous involvement of cell shrinkage results in cell apoptosis machinery triggering and progression. In some cases, apoptotic collapse may occur in the absence of cell volume changes and even follow cell swelling rather than shrinkage [30], [31]. Therefore, more convincing researches are needed to demonstrate how ENaC regulates cell volume and how cell volume perturbations induce apoptosis.

In the present study, we only detected the expression of γ-ENaC as one of the potent mechanisms. More solid evidences are needed, for example, we could silence the γ-ENaC gene or use some specific antagonists to block γ-ENaC for further verifying its involvement in the anti-apoptotic role of EETs and CYP2C23 in vitro. ENaC consists of three homologous subunits, α, β and γ. α-ENaC is mainly present at the apical domains of principal cells, whereas β- or γ-ENaC is associated with intracellular vesicles dispersed in entire cytoplasm [32]. This suggests that there may be some differences in the regulation of different ENaC subunits. Previous studies have shown discrepancies with regard to the three subunits [33], [34]. In the present study we chose γ-ENaC as the target protein because it is the predominant channel responsible for sodium absorption in rat IMCD [2], [3]. Admittedly, α- and β-ENaC may also be involved in the process. Future studies will be aimed at defining the role of other ENaC subunits in the proliferation and apoptosis of IMCD cells. Taken together our results indicated that inhibition of γ-ENaC may be one of the possible mechanisms contributing to the anti-apoptotic effects of EETs on IMCD cells exposed to hypotonic stress.

Furthermore, γ-ENaC has been described to be involved in the anti-apoptotic role of EETs, but the signaling mechanisms are still unclear. The data from our work and others indicated that EETs by CYP2C and/or CYP2J significantly promoted endothelial cell proliferation and attenuated apoptosis, which was associated with PI3K/AKT and MAPK signaling pathways [35]–[37]. Naomi Niisato and some other scientists have shown that three members of the MAPK family, p38, JNK and ERK, and the EGFR-JNK-PI3K pathway contribute to the stimulation of sodium reabsorption in response to hypotonic stress [38]–[40]. Therefore, MAPK family and PI3K/AKT pathway may be involved in the anti-apoptotic role of EETs via γ-ENaC in IMCD cells exposed to hypotonic stress. Further studies are required to illustrate the potent relationships between them.

In addition, as many other ion channels, ENaC is regulated by factors that alter gating, expression, subunit assembly, membrane translocation, phosphorylation/dephosphorylation, and residence time. Multiple mechanisms can alter one or more of these factors, including proteolysis, hormonal effects on translocation and membrane assembly, changes in ubiquitination, retrieval, and degradation, and protein kinase-mediated negative or positive effects [24]. In this study we did not probe into this aspect. Further studies are required to define the precise mechanisms on the role of ENaC in anti-apoptotic process.

In conclusion, our results demonstrated that hypotonic stress inhibited proliferation and induced significant apoptosis in IMCD cells. CYP epoxygenases-mediated EETs showed an anti-apoptotic effect on IMCD cells exposed to hypotonic stress. Down-regulation of γ-ENaC may be one of the possible mechanisms contributing to the process.

## Materials and Methods

### Experiment Reagents

All cell culture reagents were obtained from GibcoBRL (Life Technologies, Inc., Grand Island, NY). Rabbit β-actin mAb was from Cell Signaling Technology Inc (Beverly, MA). Rabbit polyclonal antibody to γ-ENaC was from Abcam Inc. (Cambridge, UK). Enhanced chemiluminescent substrate was from Pierce (Rockford, IL). PVDF membranes were from Merck Millipore (Billerica, MA). Annexin-V-FITC-PI apoptosis detection assay kits, 11,12-EET, NG-methyl-L-arginine,3-(4,5-dimethylthiazol-2-yl)-2,5-diphenyl tetrazolium bromide (MTT) and Dnase I were obtained from Sigma-Aldrich Chemical Co. (St. Louis, MO). All other chemicals and reagents were from Sigma-Aldrich Chemical Company unless otherwise specified.

### Ethics Statement

This study was carried out in strict accordance with the recommendations in the Guide for the Care and Use of Laboratory Animals of the National Institutes of Health. The protocol was approved by the Committee on the Ethics of Animal Experiments of Sun Yat-sen University (Approval Number: SYSU-2013-23). Rats were anesthetized with ether and decapitated, and all efforts were made to minimize suffering.

### IMCD Cells preparation

IMCD cells were isolated from male Sprague-Dawley rats using hypotonic lysis isolation method described previously [41]. Rats were anesthetized with ether and decapitated. The kidneys were removed and rinsed with D-Hanks solution containing antibiotics three times. The inner 1/3 medulla was dissected, minced, and incubated in 0.1% collagenase I at 37°C for 90 min. The IMCD cells were isolated by a further incubation with the above solution made hypotonic with distilled water containing 0.01% deoxyribonuclease I, and cells were digested on shaker at 37°C for 45 min. After filtering with 200 mesh filter and centrifugation, the cell pellets were washed and resuspended with medium containing 1∶1 mixture of Dulbecco's Modified Eagles Medium (DMEM) and Ham's F-12 Medium supplemented with 10% fetal bovine serum (FBS). In this way, we acquired IMCD cells with purity more than 90% [42]–[44]. Cells were seeded at a density of 1.0×10^6^ cells/well in six well plates and culture flasks. After cells reached 80-90% confluence on the 3rd day, experiments were conducted on the 5th or 6th day [45].

### Overexpression of CYP2C23 by adenovirus in IMCD cells

To achieve overexpression of CYP2C23 in vitro, we used adenoviral CYP2C23 vector, Ad-CMV-CYP2C23 (Vector Biolabs, Philadelphia, PA). In the Ad-CMV-CYP2C23 virus, expression of CYP2C23 is under the control of CMV promoter. After three rounds of amplification in QBI-293 cells, we were able to obtain a high titer of Ad-CMV-CYP2C23 (4×10^10^ VP/ml). By using the similar approach, we obtained enhanced green fluorescent protein (EGFP) vector (Ad-CMV-CYP2C23-EGFP) through a custom adenovirus service. The virus contains internal ribosome entry site of encephalomyocarditis virus between CYP2C23 and EGFP coding region, allowing both CYP2C23 and EGFP to be translated from a single bicistronic mRNA. To demonstrate the expression of recombinant CYP2C23, we transfected IMCD cells, which do not express endogenous GFP, with Ad-CMV-CYP2C23-EGFP at multiplicity of infection (MOI) of 100, and cultured the cells for 24 h. Recombinant adenovirus vectors without CYP2C23-EGFP (100 MOI) were added as control. The fluorescence intensity of GFP was observed by Inverted Flurescence Microscopy (Leica DM13000B, Germany) and quantitated by Image-Pro Plus 6.0 (Media Cybernetics) [46]–[49].

### Effects of EETs and Ad-CMV-CYP2C23-EGFP transfection on IMCD cells proliferation under hypotonic stress

The effects of EETs and Ad-CMV-CYP2C23-EGFP transfection on IMCD cells proliferation under hypotonic stress were evaluated by MTT colorimetric assay. IMCD cells were seeded in triplicate in 96-well plates (1×10^4^ cells/well) with 10% FBS DMEM/F12 medium for 72 h. When cells reached 80% confluent, the medium was changed to experimental medium with different osmotic pressure: isotonic (300 mOsmol/kgH_2_O, DMEM/F12) and hypotonic (200 mOsmol/kgH_2_O, DMEM/F12+3.4% (*V/V*) pure water). Simultaneously, the cells were stimulated with 11,12-EETs at 100 ng/ml or recombinant adenovirus Ad-CMV-CYP2C23-EGFP (100 MOI) [48], [49]. After 12 h or 24 h, viable cell numbers were estimated by MTT assay. Briefly, medium was removed and replaced with medium containing 5 mg/ml MTT and incubated for 4 h. The medium was aspirated, and the product was solubilized with dimethyl sulfoxide. Absorbance was measured at 490 nm using a microplate reader according to manufacturer's protocol.

### Flow Cytometry Analysis

Annexin-V-FITC-PI apoptosis detection assay kits were used to determine the apoptotic rate of IMCD cells. Annexin-V-FITC and PI were used for staining following the manufacturer's staining procedure. Briefly, cells were seeded at a density of 1.0×10^6^ cells/well in six well plates. After intervention described above, cells were harvested and washed with cold phosphate buffered saline (PBS), and then centrifuged. The cell pellets were resuspended to 5.0×10^5^ cells/ml. Annexin V-FITC and PI were added to cell suspensions, and the mixture was incubated on ice for 10 min in the dark. After incubation, the samples were tested. Excitation and emission settings were 492 nm and 520 nm respectively for annexin V-FITC, and 550 nm and 560–680 nm for PI (FACStar-Plus flow cytometer, BD Biosciences, San Jose, CA) [50].

### Protein Extraction and Western Blotting

The protein of IMCD cells was extracted as previously described [41]. Briefly, the media in 6-well plate was discarded and cells were gently washed three times with cooled PBS. Lysis buffer (RIPA and PMSF) was added to the cells. After incubation on ice for 30 minutes, the lysate was centrifuged at 12,000 *g* at 4°C for 10 minutes. The protein concentrations of the supernatant were determined. Lysate samples (25 μg protein/lane) were resolved by SDS-PAGE (10%), transferred to nitrocellulose membranes, and blocked with 5% nonfat dry milk in TBS-T (10 mM Tris-Cl, pH 7.5, 100 mM NaCl, 0.1% Tween-20). The membranes were then incubated with rabbit polyclonal antibody to γ-ENaC (1∶1000 dilution) overnight at 4°C, followed by peroxidase-conjugated secondary antibody for 2–3 h. The ECL system was used to visualize the separated proteins. The expression of β-actin is used as loading control.

### Statistical Analysis

All experiments were repeated at least three times. Data were analyzed with SPSS 17.0 statistical software package (SPSS, IL, USA) and presented as mean ± SD. Comparisons between groups were performed by a Student's paired two-tailed t test. Two-way analysis of variance was used to examine differences between groups, with post hoc analyses performed by Student-Newman-Keuls method. *P*<0.05 was considered to be statistically significant.
